# The Coagulation of the Blood

**Published:** 1864-04

**Authors:** 


The Coagulation of the Blood.—There is no subject which has been more fruitful in honor to British physiologists and surgeons than the study of the functions and changes of the blood and bloodvessels in health and disease. The main contributions to that knowledge which the world possesses on this subject have been made by our countrymen. Since the day when Harvey immortalized his name by his researches on the circulation, we have learned much that was unknown to that great discoverer: they are chiefly British names that adorn the roll. Hunter, Hewson, Scudamore, Gulliver, Quickett, Paget, Richardson, Bennett, and Lister, these are the men who have done much to explain the secrets of the constitution and vital qualities of the blood: Hunter, Hodgson, Cooper, Travers, Hutton, Bellingham, are names attached to those great successive advances in the surgery of the bloodvessels which have made that department of surgery remarkable in the professional history of this century. Hence great interest surrounds the subject of Professor Lister's recent Croonian Lecture before the Royal Society, which, by the consent of the lecturer, and through the kind courtesy of the Secretary of the Society, we are able to place before our readers to-day. There is no question in physiology at the present time of greater importance than the determination of the cause of the coagulation of the blood. It has always been a problem of great attraction to physiologists. It is unnecessary for us here to recite theories which have held possession of the schools, based chiefly on the conclusion of John Hunter that "coagulation is an operation of life" due to the organization of its fibrin. Until the labors of Dr. Richardson it was felt that our knowledge was excessively deficient as to the changes which took place in the blood when coagulating, and their efficient cause. His researches brought out prominently and confidently a definite result—the evolution of ammonia: and to this he ascribed, with great clearness, the phenomena of coagulation. This ammonia theory was supported by so many ingeniously devised experiments, and, moreover, squared so well with the majority of observed phenomena in the coagulation of the blood that it was eminently satisfactory to the minds of those who read the essay, and who rested content with its statements and conclusions. Since its publication, however, some competent observers have renewed their investigations, and directed especial attention to the ammonia theory. Among these counts Mr. Lister, one of the most acute and competent physiologists of the day, and especially known for his researches on inflammatory and other changes in the blood. The results at which Mr. Lister has arrived are, it should be observed, singularly opposed to the conclusions which Dr. Richardson has put forth as the fruit of his labors. Dr. Richardson, for instance, has taught that blood shed into a vessel coagulated by virtue of throwing off its ammonia, and that the more rapid coagulation on agitation was in consequence of the more rapid loss of ammonia. Mr. Lister finds that agitation of the blood within its vessels, and without loss of ammonia, produces identical effects. Dr. Richardson attributed the acceleration of the coagulation of blood under heat to the volatization of ammonia, and its retardation by cold to the imprisonment of that volatile alkali. Mr. Lister finds heat and cold produce those relative effects equally when means are taken to place the blood in an ammonia bath.

The lecture of Professor Lister presents, in short, a series of experiments and conclusions totally adverse to the theory which Dr. Richardson propounded on the strength of his laborious research, and supports a physical explanation of the phenomena. The oration is one which must command attention by its singular lucidity and the logical power with which many most ingenious and delicate experiments have been employed to furnish materials for a continuous argument which seems to carry conviction. Dr. Richardson may find replies to the objections of Professor Lister; but the value of the investigation must in any case be very great, for it produces irrefragable evidence of physical relations of the blood which are of the highest importance to the pathology of inflammation, the formation of emboli and clots, and which will form the basis for advance in blood-physiology and pathology.

